# Philadelphia Hospital

**Published:** 1838-11-21

**Authors:** 


### CLINICAL LECTURES.

### PHILADELPHIA HOSPITAL.

The clinical course, in this hospital, commenced on Saturday, November 10th. Dr. Gibson opened with some general remarks on the advantages of the mode of instruction in operation in this hospital, and originated, as he said, by himself. To lecture actually at the bed-side, in the ward, we had long since discovered to be a very inefficient means of teaching any considerable body of students. However well this mode might answer with a class limited to a dozen or twenty, it would not be successfully employed, when the number of pupils amounted to some hundreds, as in the present instance. He had therefore adopted the plan of having the patient introduced into the amphitheatre, where he might be seen by every individual present, to whom whatever remarks the case might suggest, would likewise be distinctly conveyed.

A patient was now introduced with a dislocation of the os humeri into the axilla, of eighteen days standing. The man had had a similar dislocation upwards of two years ago, caused by a fall from a hay mow, upon his hand. It was restored in the usual way, but had left a disposition to a return of the accident: the rupture in the capsular ligament remained a permanent slit, through which a slight cause, such as a preternatural elevation of the arm, would tend to make the head of the bone slip. In one instance, he had reduced a luxation *fourteen* times, in the same individual. These secondary luxations are commonly easy of reduction, from the same cause which induced the liability to their occurrence. A first luxation is much more difficult to reduce; and experience has proved that certain circumstances may arise, after the lapse of time, which renders it an exceedingly dangerous operation.

The reduction of the luxation was now attempted by simple extension with the hand, and the heel in the axilla. This was kept up for seven minutes, without success. Extension was afterward effected by a towel bandaged to the fore arm. Dr. Gibson stated that the luxation originally into the axilla, had been now converted into one forward under the pectoral muscle. It was reduced in two minutes, by the means mentioned. Owing to the wasting away of the deltoid muscle, the accident having been of near three weeks standing, the roundness of the shoulder was not entirely restored.

Dr. Jackson followed Dr. Gibson. He stated that the course of lectures on clinical medicine, would be given by himself, in conjunction with Dr. Gerhard. Before taking up his particular subject, he wished them distinctly to understand the position in which he and the other lecturers stood before them. They were not there as professors of any school, but they had been selected as physicians of Philadelphia, and not from their connection with this or that college, or university. The institution of clinical lectures in this hospital, was the result of a voluntary organization on the part of the physicians and surgeons. They had no peculiar interests to serve from this course, nor did they derive from it any direct pecuniary advantage. He had been lecturing there for fifteen or twenty years, in which period the managers had, he supposed, received some twenty thousand dollars from this source, and yet he and his colleagues had not received so much as a vote of thanks. On the contrary, the managers seemed to think that they conferred a favor by permitting them to lecture, not that they received one from their gratuitous exertions.

The next point to which he should call their attention, was the nature of the instruction which they came there to receive. Clinical differed from didactic instruction, in its demonstrative character. When properly conducted, its object was not to give the history, general character, and symptoms of a disease, but to confine the attention of the class to the particular case under notice. That case was the sole subject of examination, and the views of the lecturer were to be given with regard to the diagnosis, prognosis and treatment of it. But, for this purpose, it was necessary that the case should be followed out by the student from day to day. This, the distance of the hospital from town rendered incompatible with their other pursuits, and. hence, it was impossible to give them a purely clinical course. It must be necessarily of a mixed character, consisting of a general lecture, upon a given subject, to be afterwards illustrated by such cases as the hospital afforded. He could not but regret this unfortunate drawback upon their clinical instruction, and that they had not a hospital better situated, and under the management of the profession. He considered one of the greatest wants of the day to be a hospital under the sole control of the medical profession, which he hoped, before a very long time, to see established.

The next point to which he should allude, was the *character* of the patients who entered this hospital, and consequently of the instruction which was given there. They would not often witness acute diseases in the wards of the hospital; the great mass of the cases there were chronic, and might, perhaps, at first sight, seem to them of inferior interest. But this was a mistake; they were of greater value to them than many with which they might be at first more struck. They were the cases of every day occurrence; they were met with daily by the physician, and, when they came to practise themselves, they would understand their value. They were of vastly more importance than the rare and uncommon cases; and they would find, that of the first class of cases this hospital afforded an abundance.

In the examination of the cases which came before them, Dr. Jackson said that he would chiefly dwell upon the diagnosis, or the mode of determining the peculiar character of the affection. This was the most important point connected with the examination of disease. Before they undertook to cure, they must know what they had to cure. A knowledge of diagnosis was based upon a knowledge of anatomy and physiology. They could not know when an organ was diseased, unless they knew what were its structure and function. Let them make themselves thorough masters of anatomy and physiology, and they would have no difficulty in understanding the derangements of organs and functions, of which they knew the healthy conditions. Diagnosis includes many important points. Among them are the causes of disease, and the symptoms by which it is known.

It is our object, to-day, to present you with a number of cases of different varieties of disease, affecting various organs of the body, and all characterized by appropriate symptoms. You will then acquire a general idea of diseased action, and will be better qualified for entering upon the study of the individual affections of which we shall speak at greater length in a subsequent part of the course.

The first class of affections, which we present to you, are the cerebral diseases. You have here two striking cases of hemiplegia, following apoplexy; and one of chronic meningitis. Last week we had several interesting cases of inflammation of the brain, but we have none at present in the wards. One of the cases of hemiplegia occurred in a lunatic in the asylum; the patient was found one morning speechless, and in a state of almost complete stupor; after free cupping and purging, he recovered from the stupor, but remained in the condition in which you now see him. His left arm and left leg are completely paralyzed, and fall by their own weight; the skin on that side of the body is completely insensible, and its temperature approaches more nearly to that of the external air, than that of the right side. His mouth is slightly drawn to the right side, in consequence of the paralysis of the muscles of the left side of the face. These symptoms are permanent, the only change which has occurred in them, since the admission of the patient, is a certain degree of stiffness in the joints, which, in part, results from their having been at rest for so long a time, and, in part, from a real spasmodic contraction of the muscles. The pathological lesions connected with this series of symptoms, are equally constant; we know from experience that they consist in hæmorrhage into the right hemisphere of the brain; that is, the one opposite to the paralyzed limbs. A clot of blood has been effused into the optic thalamus, or corpus striatum, and is in gradual process of absorption. It will finally be removed, and will be replaced by a mass of cellular substance, or by an organized cyst, lined by a regular serous membrane. The stiffness is a subsequent symptom, occurring after the hæmorrhage, and depends upon inflammation and softening of the cerebral substance immediately surrounding the clot. The same patient offered us, last week, a change in the character of the symptoms; he became dull, almost relapsed into his former stupor, and his face was suddenly flushed. The brain then became the seat of active congestion, or a sort of false apoplexy, or "coup de sang," as it is called by some of the French writers. This sudden congestion required active treatment, and quickly disappeared after a free cupping and purging.

The second patient represents the same form of disease in a milder degree. He was at work in a blacksmith's shop, more than a twelve-month ago, when he suddenly found that he was incapable of raising his right arm; he had had no headache, vertigo, or other premonitory sign. Ever since that period he has walked with difficulty, and has been quite unable to use his right arm. He speaks in a thick and faltering tone, but his articulation is much more distinct than that of the patient just presented to you; you may observe that the paralysis is less complete in the leg than in the arm; this difference is usually observed in hemiplegia. Some patients lose the use of one arm, but can still walk, or if the hæmorrhage be less abundant, there is a mere numbness and imperfect motion of one arm without entire paralysis. The countenance of this patient is scarcely distorted, there is merely a dull, stupid expression, dependent upon the paralysis. He will recover almost entirely; the last patient is incurable. This difference in the prognosis depends, as you will afterwards perceive, almost entirely upon the quantity of blood affused. If it be small in quantity, the wound in the brain may be entirely healed; if the quantity be very considerable, an incurable mischief is done to the substance of the brain, and the patient never recovers.

The third case of cerebral disease, is offered by the third patient, who is labouring under chronic meningitis. He received, about thirty years ago, when a lad of sixteen or seventeen years of age, a blow upon the head. Since that period he has been subject to dizziness and headache; his mind has gradually become weaker, and he became conscious of an inability to reflect with his usual clearness. About three months since, he entered the ward with the symptoms just enumerated, and also laboured under commencing paralysis; he staggered in his gait, and the strength of both arms was considerably impaired. There was a little difference on the two sides of the median line, but still the paralysis was not confined to either; there was not hemiplegia, as in the other cases. Within the last few days only, the intellectual symptoms, if we may use the expression, have increased. He has become quite delirious, talks wildly and incoherently, leaves his bed at night, and is permanently insane. The disease has, in the present instance, followed a blow upon the head, but it does sometimes occur without any obvious external cause. From its frequency among lunatics, it is sometimes called the paralysis of the insane, and you will find that it is described very carefully by Calmeil.

We next offer you examples of thoracic disease. The first patient is, you perceive, a young woman, who labours under pericarditis. Her countenance is pale, with a bluish tint at the lips and nostrils; she breathes with difficulty, and has a short dry cough. These symptoms are not peculiar to diseases of the heart, they may belong to those of the lung; we distinguish between them by the physical signs. In the present instance, there are prominence of the præcordial region; flatness on percussion, as you may perceive by listening attentively while I tap lightly on my finger laid over the heart, and the signs derived from auscultation, to which we need not refer at present

As examples of diseases of the lungs, we offer you several cases of phthisis; you perceive that each of these four patients has cough, fever, emaciation, and expectoration of mucous, or of muco-purulent matter. The young woman has more fever than the others, and presents upon her cheek a circumscribed redness, which arises from the fever in this disease, as soon as it assumes the character of hectic. These are all chronic cases of diseased lung, and we select them in preference to cases of emphysema, or chronic bronchitis, because their symptoms are more intense. We have but few examples of acute disease of the lung during the present mild weather, and it is better for us to wait until cases which are well characterized enter the hospital.

Abdominal disease is usually recognised by signs, as distinctive as those of the other cavities of the body. We present to you several instances of the kind.

Of dysentery, we have numerous examples during the summer and autumnal months. The commencement of the colder weather has rendered these cases comparatively rare. The patient now before you is already convalescing; nevertheless you may still observe some of the traces of the disease in his countenance. He is haggard, his skin is of a dusky hue, there are deep furrows on each cheek, and the corners of the mouth are drawn downwards. There is nothing of the disturbance of the intellect, the altered eye, or of the lesion of mobility or sensibility, which you remarked in the cerebral cases, neither have you the dyspnœa, the flushed cheek, cough, or expectoration, noticeable in the cases of disease of the thorax. The expression about the countenance, consisting in the drawing down of the corners of the mouth, is in some degree dependent upon the nausea which the patient suffered during his illness; in which he presented the common complication of gastritis, together with the dysentery. Although the disease has in a great measure ceased, we still have the traces of it very evidently remaining behind.

The next two patients offer other indications of severe abdominal diseases. One is labouring under an enlargement of the liver; his mind is so much affected as to render him quite insensible to our remarks. The history of the case is very imperfect, in consequence of the feeble condition of his memory and intelligence since his admission. We learned, however, that he had been ill with diarrhœa for nearly six months, during which period he had scarcely been sober. The disease of the liver arises, therefore, from two causes, either of which is sufficient to produce it—the chronic irritation of the colon, and habitual intemperance. At his admission, there was already a decided yellow tint of the skin; but in a day or two afterwards it became much more marked than it had previously been. About the same period there were symptoms of delirium tremens, which obliged us to transfer the patient to the cells. These symptoms soon subsided, but the intelligence of the patient was not restored; his memory was entirely lost, and he presented a slight form of delirium, with muttering and stupor, of a similar kind to that which is frequently observed in the latter stages of meningitis. We did not, however, regard the case as one of true inflammation of the membranes of the brain, because we had already sufficient evidence of disease of the liver from the enlargement and tension of that organ, as well as the jaundiced hue of the skin. We were aware that the cerebral symptoms were a frequent, if not a regular, accompaniment of the severer grades of jaundice, and that they arose, in all probability, from the admixture of bile with the blood. An analogous kind of delirium occurs also in renal disease, and probably depends upon a similar cause—that is, the mixture of the principle of urine with the circulating fluids. The case is a remarkable one, and well worth your careful study.

The last case of abdominal disease which we shall show you to-day, is offered by the patient who is labouring under ascites. He was about a year since in the surgical ward; an operation was performed for the removal of a fungus hæmatoides from the head. The wound healed without any untoward accident. Diarrhœa had supervened during his residence in the surgical ward; and after the cure of the external disease, he was transferred to the medical ward, where the diarrhœa was speedily arrested by very simple treatment. The patient became well enough to leave the hospital, and continued at ordinary labouring work for several months. His health began to fail him about eight weeks before his entrance, and within the last four weeks he suffered much from wandering pains in the back and abdomen. The swelling commenced about three weeks since—that is, it became considerable enough to be perceived by the patient about that time. I shall not detain you upon the symptoms of the disease, but will merely point out the haggard, contracted countenance, and anxious expression of the patient. The obvious disease is, of course, the effusion of liquid in the abdomen; which, as you perceive, is enormously distended, and yields, on tapping it lightly, an evident fluctuation. By resorting to another means of examination, percussion, we are not only able to prove that the peritoneum is distended with liquid, but to point out the line of division between the liquid and the gas contained at the upper part of the abdomen. The latter means of examination is of much importance in practice.

These cases are all sufficiently characterized, and may give you some idea of the mode of examination, and of the various expressions of diseases; all minute details are purposely omitted, and would, of course, be misplaced in a general lecture.

At the conclusion of the lecture, a demonstration was made by Dr. Gerhard of the pathological changes observed in a case of pulmonary consumption. The disease had been of several years duration; but the immediate cause of death was diarrhœa, which had occurred some weeks previously to the termination of the disease. The mucous membrane of the colon was ulcerated throughout its whole extent, but there were no tubercles in any portion of the intestinal canal. Tubercles were very numerous throughout the whole of the lungs, and at the apex of the left there was a flattened cavity, an inch and a half long, extending across the lung, at a little distance below the stomach. This cavity was lined by a hard membrane, and evidently of long standing, and admitted air with so much difficulty, that cavernous respiration and pectoriloquy did not exist until the patient was made to cough very forcibly.

